# Reviews and Book Notices

**Published:** 1878-03

**Authors:** 


### Reviews and Book Notices.

THE ACTION OF MEDICINES. By Isaac Ott, A. M., M. D., formerly Demonstrator of Experimental Physiology, University of Pennsylvania, with twenty-two illustrations, pp, 168. Philadelphia: Lindsay & Blakiston, 1878.

In this small volume the first hundred pages treat of the methods of studying the action of medicines, while the last sixty pages are devoted to the physiological history of the separate drugs. In a very systematic manner the author details in the first chapter the methods of selecting and handling animals and preparing them for the experiments. A lucid description of the instruments and apparatus necessary is also given. This is done in precise language, though not always elegant English, carefully avoiding diffuseness. But on the other hand some paragraphs suffer by too fragmentary a treatment; for instance, the page on physiological anatomy is entirely too sketchy to be a guide to the student, and unfortunately this reproach applies to many a page in the book. A second chapter on the action on the nervous system is excellent as far as it goes, but it might be more complete. By far the most satisfactory part of the book is the following chapter on the circulating apparatus. The methods of studying the action of poisons on the different cardiac and vasomoter nerves, and the observation on the excised frog's heart are detailed in a masterly manner.

Notwithstanding the omissions and defects, the first part of the book cannot be too highly recommended to the American public. It is the first attempt of its kind in this country. The author insists, and very justly, that the study of therepeutics ought to be more than crude empiricism, and the methods he describes are really the roads which, if followed, will eventually lead to a science of therapeutics.

As regards the second part of the book, the less we say of it the better. The author shows by his quotations his extensive knowledge of the literature of the subject, and perhaps the references he gives are the most valuable part of the chapter. The physiological history of the separate poisons is unsatisfactorily short, while the explanations of the curative effect are not always happy instances of theoretical deductions. Since such comprehensive works as those of H. C. Wood and others can be had for reference on the effects of individual drugs, we cannot doubt but that the book would have gained by the omission of the last chapter.

H. G.

Obstetrics Reduced to Questions and Answers. By Mrs. L. H. Corr, M. D. D. B. Cooke & Co., Chicago.

This book is a treatise on the obstetrical art in a novel form, and professes to furnish all needful information to the student in small and practical compass.

For the most part this promise is ably fulfilled, a plain statement of modern views being afforded as an answer to every question without discussion of mooted points. While the method of treatment would be unsatisfactory to the advanced thinkers, it is well adapted to the inculcation of first principles. The medical student about presenting himself for final examination does not care to be encumbered with *impedimenta*, nor when engaged in his first obstetric offices, can he find comfort in a variety of opinions.

The subject of embryology is especially well managed in this respect, and the text is amply illustrated here, as in other parts, by appropriate diagrams.

In the management of natural labor the maxim "meddlesome midwifery is bad" seems to have influenced the author to such an extent as to lead to the conviction that she leans toward the conservatism of the lecture room, rather than to the procedure of experience. No fault can be found, however, in the more precise formulæ for the treatment of difficult and complicated labor. This subject is as well presented as possible in such a work.

The management of the so-called "puerperal condition" admits of more discussion than is here allowed, and the author seems to have taken for granted methods, the safety of which, to say the least, is not conceded by many reputable teachers.

Excepting the minor inaccuracies, which a second edition will undoubtedly remedy, the book presents a creditable appearance, and does honor to its compiler and publisher. It deserves a place in every student's library. H. W. J.

Nurse and Patient and Camp Cure. By S. Wier Mitchell, M. D. Lippincott & Co., Philadelphia, 1877.

An excellent little work full of suggestion and sound advice to both physician and patient, and also to the family and family nurse. The object in putting forth the paper on "Nurse and Patient" is to supply some omissions observed in Miss Nightingale's book on nursing, pointing toward the evils of amateurs, and the pernicious effects resulting both to patient and nurse between whom exist strong ties of love or near ties of kindred. As a rule the best nursing is paid nursing, and the worst very often that which comes from the family. Too often however financial circumstances preclude the idea of hired nursing, and this work must fall upon some member of the family. In this case the doctor extends some wholesome advice relative to the safe carrying of the newly imposed burden so as to give to the patient all needful attention, and at the same time protect the nurse from making of herself, through "that strange feminine mood of sacrifice" a needless martyr, thereby, ultimately, adding another invalid to the family.

The main purpose of the paper devoted to "Camp Cure" is to insist upon the great value to people in and out of health—particularly brain working people living in large cities, and victims of civilization—of returning occasionally to some form of barbarism. "Civilization has hurt, barbarism shall heal." His vivid discription of camp life and the beautiful presentations in word picture of the scenery of the upper Superior country and the lakes and woods of Maine given in his own fascinating and inimitable style almost bring to the reader the "breath of the wood" and the sound of the waterfall and the splash of the oar: and one begins to feel a growing respect and salivary longing for the prejudicial onion which can be eaten in camp with impunity by the most dyspeptic. The weary "professional," the merchants, the manufacturers, dealers in money, etc., who through successive years, have been held to their severe and steady labors, and who experience, as they must as a result, the prostation and fag and relaxation which the springs peculiar to our climate bring, would do well to try the camp cure in place of the murky watering places and fashionable resorts which entice only to deceive.

One with aching and weary brain, with ears filled with the cities' din, and a growing perception within and around of approaching summer's heat, cannot read this little work without being seized with a desire to shoulder rod or gun and a knapsack, to "change," turn his back on civilization and take a plunge into such delicious barbarisms as are here briefly sketched.

S. F. B.

On the Uses of Wines in Health and Disease. By Francis E. Anstie, M. D., F. R. C. P. Late physician to the Westminster Hospital, and editor of the *Practitioner.* Reprinted from the *Practitioner.* London: MacMillan & Co., 1877. pp. 74. Chicago: Jansen, McClurg & Co.

This little book should be purchased by every physician, and in fact by everybody who wishes to obtain some sound, unbiased information on the use of wines. It presents the various sides of the question in the calm spirit of scientific exposition and in the elegant language of an experienced and refined author.

If the knowledge obtained in this little treatise was more widely diffused among all classes of the American people, there would be less occasion and less need for temperance movements which, it seems, must break out at regular intervals with the same necessity as the periodic upheavals of a volcano follow the accumulation of immense volumes of gas.

State Regulation of Vice. Regulation Efforts in America. The Geneva Congress. By Aaron M. Powell. New York: Wood & Holbrook, publishers, 1878; pp. 127.

The question as to the best way of dealing with the so-called social vice, is strongly occupying the minds of both lawyers and physicians; the former chiefly because of the danger involved for the morals of society; the latter on account of the propagation of the venereal diseases. An international congress was held in Geneva, Switzerland, in September, 1877, to promote the abolition of State regulation of prostitution. The little book gives an account of the proceedings and conclusions of this congress, and also describes the various attempts which were made in American cities to introduce this European system of regulating the trade of prostitutes by State laws. The author is a pronounced enemy of State regulation, and takes a great delight in showing up the utter inefficiency of this system by statistical abstracts from the police records of Paris, where the system has been in force these many years. These records prove that during this time prostitution and syphilis have not only not abated but increased in a very appreciable degree. This result cannot surprise any one who knows that all attempts so far made toward arresting the propagation of syphilis were too one sided to be efficient. A system which limits the medical examination to the prostitute women and leaves their male visitors unexamined, has as much protective power as the plan to bolt the stable door after the horse is stolen.

